# Chest Ultrasonography in Modern Day Extreme Settings: From Military Setting and Natural Disasters to Space Flights and Extreme Sports

**DOI:** 10.1155/2018/8739704

**Published:** 2018-03-15

**Authors:** Francesco Feletti, Viviana Mucci, Andrea Aliverti

**Affiliations:** ^1^Department of Diagnostic Imaging, AUSL Romagna, S. Maria delle Croci Hospital, Ravenna, Italy; ^2^Dipartmento di Elettronica, Informazione e Bioingegneria (DEIB), Politecnico di Milano, Milan, Italy; ^3^Antwerpen University Research Centre for Equilibrium and Aerospace (AUREA), Antwerp, Belgium

## Abstract

Chest ultrasonography (CU) is a noninvasive imaging technique able to provide an immediate diagnosis of the underlying aetiology of acute respiratory failure and traumatic chest injuries. Given the great technologies, it is now possible to perform accurate CU in remote and adverse environments including the combat field, extreme sport settings, and environmental disasters, as well as during space missions. Today, the usage of CU in the extreme emergency setting is more likely to occur, as this technique proved to be a fast diagnostic tool to assist resuscitation manoeuvres and interventional procedures in many cases. A scientific literature review is presented here. This was based on a systematic search of published literature, on the following online databases: PubMed and Scopus. The following words were used: “chest sonography,” “ thoracic ultrasound,” and “lung sonography,” in different combinations with “extreme sport,” “extreme environment,” “wilderness,” “catastrophe,” and “extreme conditions.” This manuscript reports the most relevant usages of CU in the extreme setting as well as technological improvements and current limitations. CU application in the extreme setting is further encouraged here.

## 1. Introduction

In the past two decades, the prehospital treatment in extreme settings has greatly advanced [1, 2]. Extreme settings are known for posing additional challenges to medical operations. Extreme settings are described as situations characterized by austere nature and specific inherent threats encountered (e.g., avalanches). These can vary from war scenarios to natural disasters, high-altitude missions, and extreme sport scenarios (e.g., deserted areas, savage seas, and high mountains) [3]. Medical operations in extreme settings usually require the need for integration of medical and rescue skills, as well as the demand for prehospital medicine and expanded care protocols [3, 4].

The military field has implemented over the year specific extreme condition infield trauma management techniques [5]. In addition to this, they have developed specific protocols and procedures including logistical plans for medical evacuation of casualties and for climatic control for medical devices [6, 7].

Extreme environments are also considered remote and adverse locations where most extreme sports are practiced. Extreme sports have exponentially grown in popularity for the past 40 years [8]; these activities are often performed in austere environments (tumbling rivers, canyons, and glaciers) far away from medical assistance [9] and difficult to be reached.

Similarly, complex medical assistance is also present when considering space missions. Commercial orbital and suborbital space flights are becoming a solid reality, since Red Bull Stratos and StratEx Space Dive have opened a new era in the last year [10, 11]. In addition to these, other extreme medical settings are going to be present in the next decades due to climate change. With regard to this topic, climate change is leading to a change in distribution of infectious diseases and an increased risk for unexpected weather events capable of destroying infrastructures and creating a wilderness, isolated environment in a previously urban context [3, 12].

An efficient tool to improve medical management in the above described extreme setting is ultrasound. Clinicians in the extreme setting are challenged by the uncomfortable, often unsafe, and dangerous locations, with limited supplied and appropriate equipment.

Chest ultrasonography (CU) is a versatile, accurate diagnostic, low-cost, nonionising tool. It is based on noninvasive techniques [13] that can be easily performed in the field, through its portable device version. CU can be used as the tool for quick and accurate diagnosis prior to reaching organized medical facilities or during transportation [14–17]. Moreover, CU can also assist in resource allocation, decisions, screening for conditions, and patient management [18].

Given the multifunction of CU, this manuscript focuses on the usage of CU when dealing with respiratory failure as well as traumatic thoracic injuries in extreme settings. Thoracic injuries and respiratory failure are possible life-threatening conditions when considering extreme settings Chest injuries are representing about a quarter of the causes of death related to traumas, and they are commonly reported in extreme settings [1, 19]. Chest imaging is essential when dealing with critically ill patients, and CU has been used as a role in the prevention, assessment, and treatment of many traumatic and nontraumatic emergencies [20, 21]. Indeed, CU is a type of examination that offers more details and more sensible data compared to the traditional radiological exam for the diagnosis of many conditions, including pneumothorax, pleural effusion, and lung contusion [22–24]. In addition, CU is able to guide the decision-making process and to assist interventional procedures [21, 25].

This paper aims to review the scientific literature about the usages of CU in extreme settings, in order to elucidate the applications of this imaging method in extreme emerging situations. Secondly, we performed an overview on the current limitations of commercially available CU devices, and we proposed new technological solutions to address them. Lastly, operator-training models and trainings for advancing the use of CU in all extreme conditions are discussed here. Based on the current review, we hypothesized that, in the future, the use of CU in extreme settings will increase.

## 2. Methods

This review was performed using the following database: PubMed and Scopus. We used the following search term categories in combination: “chest sonography,” “ thoracic ultrasound,” and “lung sonography,” in different combinations with “extreme sport,” “extreme environment,” “wilderness,” “catastrophe,” and “extreme conditions.”

Papers were also included using the ancestry approach in order to include anecdotal but relevant reports.

## 3. Results

The scientific literature related to the use of CU in extreme settings is still sparse Table 1, it is possible to observe the principle applications of CU and its usages reported in the literature.

In Table 2, conditions for which assessment and treatment CU have been proposed in the wilderness setting are given.

## 4. Discussion

### 4.1. CU for Traumas

In trauma, CU provides immediate yes/no diagnostic answers to the operators during emergency treatment. Consequently, CU is usually integrated in the focused assessment sonography for trauma (FAST) protocol with the name of extended FAST protocol (e-FAST) or chest abdominal FAST (CA-FAST).

The latter in addition to the upper right and left abdomen, cardiac and pelvic views of the FAST examination, includes also pleura, lung, and pericardium assessment [24]. Repeatability is of particular relevance when considering a trauma. For example, the repeated assessment of pericardial effusion allows a prompt diagnosis of tamponade, a life-threatening condition, also reported in the extreme sport scenario [42].

In addition, many wilderness casualties can be assessed without the help of X-ray examination, but just by using CU. More in detail, CU allows to easily diagnosing chest wall injuries, including muscular lesions and hematomas and bony cage fractures and their complications [17, 18, 43–45].

### 4.2. CU in Nontraumatic Conditions

In addition to the assessment of traumatic injuries, CU has been shown to be of great advantage during the assessment of pulmonary edema (PE) in high-altitude missions as well as after apnea diving (Table 1).

Pratali et al. reported that, among climbers with the usage of CU, an inverse correlation between O_2_ saturation and the progressive increase of B-lines during ascent sports [35] was detected. On the other hand, in a study by Frassi et al. on breath-hold deep divers assessed by means of CU, an increase of B-lines within 10 minutes after immersion in 45% of the participants was documented. The change, only in 28% of the cases, was associated with clinical symptoms [32]. Given those observations, the usages of CU reported here clearly show that this technology allows detecting even subclinical episodes of lung fluid accumulation, as the one reported in extreme sport participants. CU also proved high diagnostic accuracy for the diagnosis of pneumonia and chest wall infections in extreme low-resource settings [17, 18, 44, 46]. The usage of CU allowed to identify and to promptly treat vulnerable subjects on the spot [47].

### 4.3. CU Assistance in Resuscitation Manoeuvres and Interventional Procedures

In extreme settings, CU may additionally assist interventional procedures as part of resuscitation manoeuvres and patient stabilization, including positioning of catheters and tubes, pericardiocentesis, needle thoracostomy, and cricothyroidotomy [21, 25, 48]. In particular, CU has been shown to accurately and quickly evaluate the position of the endotracheal tube and its malpositioning in wilderness settings, including during air medical transports, where capnography is not available and auscultation is impossible due to noise [48].

During the resuscitation evaluation process, the CU usage is able to provide insights about hemodynamic monitoring, drug administration, and potential building up of fluids and blood products [21], since it is also used to estimate the intravascular volume status and to determine the opportunity of CPR continuation [25, 49].

Assessing intravascular volume status by measuring the IVC diameter has been previously shown to be particularly important during resuscitation evaluation. Some examples of the implementation of this technique have been described with young healthy military members, as well as extreme sport participants, since they generally have a large cardiovascular reserve and may be more severely volume depleted than their initial vital signs may suggest [44].

### 4.4. CU in the War Setting

The US military has pointed out that many clinicians are forced to “do more with less” in austere and deployment environments [6].

Iraq and Afghanistan wars were the most recent wars that required trained physicians and emergency medical crew. While during the First Gulf War, a mature multiservice medical system was never established during the latest events; hence, such a team was implemented [44]. Today, the emergency team plays a key role in the combat environment, due to their unique trainings for critically ill and traumatically injured patients [50].

Emergency ultrasonography usage for treating trauma was quickly embraced by the military, after 1997, when such devices became smaller and portable. Indeed, a small handheld ultrasound device that was light, portable, durable, and battery powered was needed only in such a way CU could be used on the battlefield. Nowadays, with the new technological advancement, such a technology exists [44, 50].

Throughout the years, the military emergency teams gained more experience with emergency ultrasonography, creating what is now named a tactical use of the ultrasound technology. Tactical ultrasound is the use of emergency ultrasound to guide decision-making in the diagnosis, treatment, and disposition of patients when resources are scarce and testing is severely limited [44]. CU is included in tactical ultrasound in the form of e-FAST protocol [44].

### 4.5. Chest Ultrasonography in Weather Disasters

CU has been shown to be a useful diagnostic tool to be implemented outside the hospital setting such as for mass casualties after natural disasters, including earthquakes, tropical storms, hurricanes, tornadoes, volcanoes, avalanches, mudslides, and floods [51].

Extreme weather conditions are likely to become more and more common due to global warming in the next decades [52].

CU has already been successfully implemented during the Armenian earthquake in 1988 and the Wenchuan earthquake in 2008. During the Armenian disaster, three major cities were affected, and more than 100 villages were destroyed [50]. Out of the 400 patients who were screened sonographically, 304 demonstrated no clinically significant pathology. In almost 25% of the cases (96 patients), a pathological condition was diagnosed by the use of ultrasound [2]. In their prospective observational study about the use of a portable ultrasound in the Amazon Jungle, Blaivas et al. [20] suggest the use of CU in either blunt or penetrating thoracic trauma in remote settings. It is mainly used to diagnose or rule out hemothorax, pericardial effusion, fractures, diaphragmatic rupture, and pneumothorax, as well as a guide to obtaining vascular access.

### 4.6. Chest Ultrasound in Space

Space is another extreme setting where CU has been shown to increase medical performances [53]. Today, ultrasound is the only imaging tool available in space on board of the International Space Station [53]. At this stage, CU is mostly used for monitoring the drastic physiological changes occurring in microgravity, as well as used a primary diagnostic tool [53].

The first ultrasound system in space was flown on Salyut 6 and 7 to study cardiovascular changes, such as heart chamber sizes and left ventricular systolic functions [53]. The use of ultrasound has enabled to increase the knowledge on fluid shifting occurring during space adaptations.

Back pain, contusion, and strain are common complains among astronauts [53]. Ultrasound technology was able to detect that, during microgravity exposure, the space between the vertebrae increases [53], possibly explaining why complains of back pain are common after space flights.

**Figure 1:**
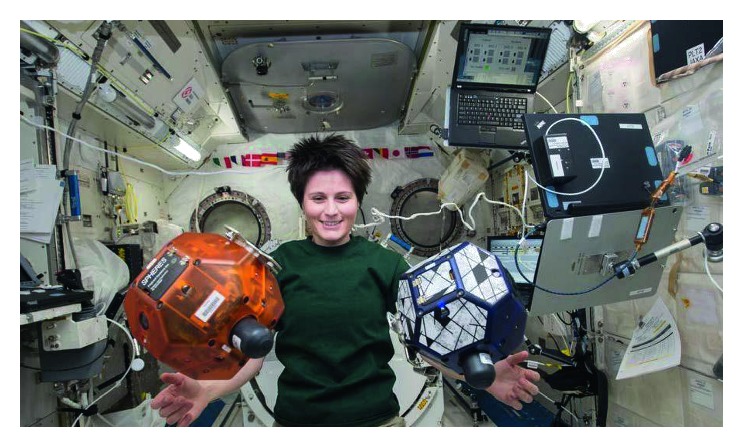
ESA astronaut S. Cristoforetti in microgravity onboard of the International Space Station.

Ultrasound in space can be used for many purposes including the assessment of tendon, ligament, and bursa diseases, as well as during traumas. The risk of catastrophic and trauma events is generally low in space due to microgravity (Figure 1); however, penetrating traumas can occur, especially during extra vehicular activities (EVAs). As a result, models on Earth using parabolic flights have assessed the potential use of FAST examinations for space missions. Some common thoracic conditions may be considered as particularly severe in space; for example, pneumothorax, in which treatment will require a chest tube placement. This is considered as a mission-terminating event [54]. Therefore, the use of CU in such an event will be crucial for the decision-making process.

**Figure 2:**
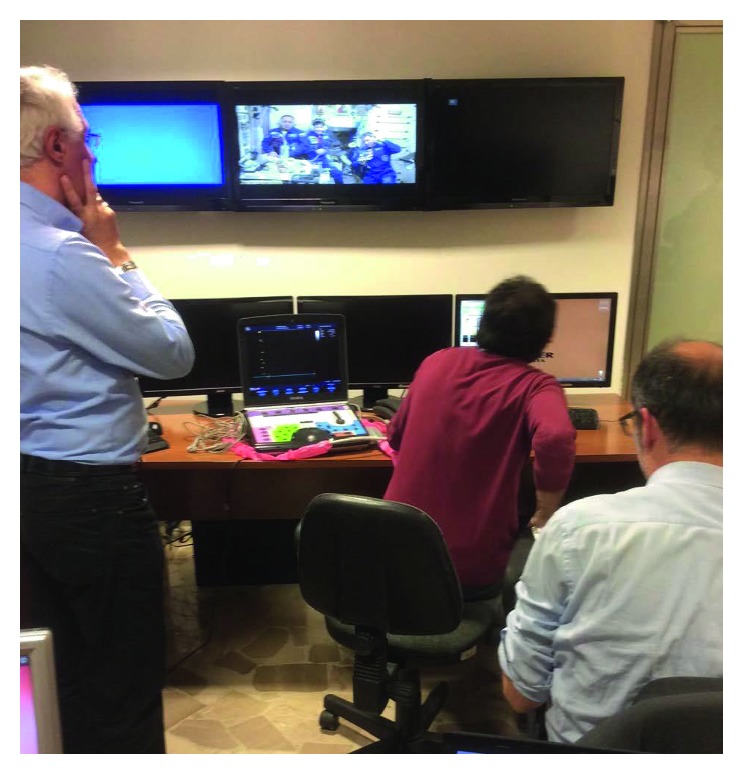
Mission control guiding the astronaut Cristoforetti and the other crewmembers to use the Vivid q CU device during the space mission Futura onboard of the International Space Station.

One study carried out in ISS analyzed the ability of nonphysician crewmembers to obtain ultrasound images [2]. In this study, crewmembers underwent limited ultrasound training before their mission. The ultrasound examination was conducted onboard of the ISS and transmitted via satellite to a radiologist at NASA Mission Control Centre. The radiologist viewed the images and instructed the crewmember on how to adjust the machine, its settings, and the probe placement. This assessment concluded that crewmembers could successfully perform ultrasound and provide via telemedicine relevant data to the flight surgeon [2]. Ultrasound training programs performed for astronauts and cosmonauts allowed the usage of CU as an easy tool to assess injuries in real time with remote assistance (Figure 2).

**Figure 3:**
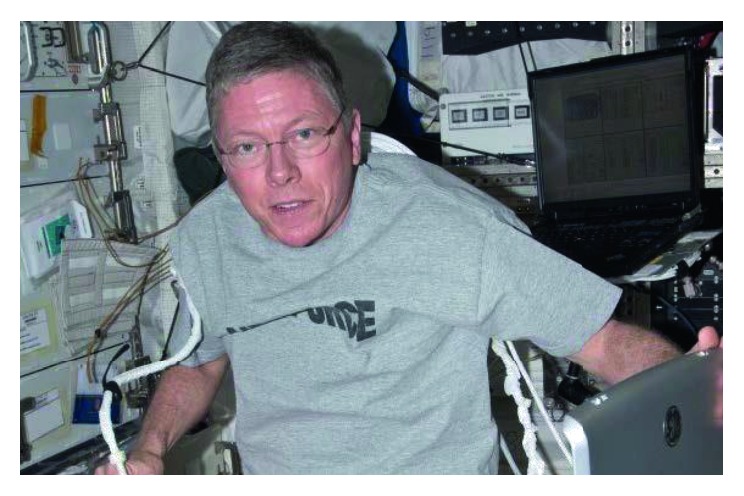
American astronaut Michael E. Fossum working with CU onboard of the International Space Station.

**Figure 4:**
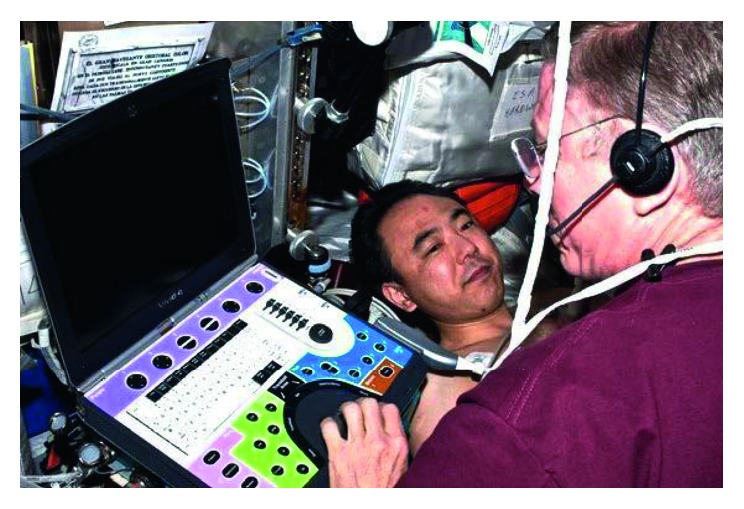
American astronaut Michael E. Fossum assessing the Japanese astronaut with CU device onboard of the International Space Station.

For these reasons, CU will continue to play a crucial role as a diagnostic method and treatment tool for medical contingencies in space (Figures 3 and 4) [53].

### 4.7. Chest Ultrasonography in Extreme Sports

The so-called extreme sports are a group of sporting activities where a likely outcome of a mistake or an accident is death due to the practice in adverse environments or to extreme psychophysical performance required [8, 9, 55]. At the moment, the literature describing or reporting CU in extreme sports is scarce. Nevertheless, the usefulness of this imaging technique in specific extreme sports, namely, skateboarding, skiing, apnea diving, climbing, mountaineering, and Ironman races, has already been established [32, 33, 35–37, 45]. Extreme sports are often practiced in remote locations, and evacuation of participants after trauma may require complicated terrestrial rescue operations or the use of helicopters or specific watercrafts. Potentially life-threatening conditions may be quickly assessed with CU directly on the scene without any delay in the evacuation [42]. Even if CU usage in extreme sports compared to other wilderness extreme fields seems limited, the use of CU for chest trauma in extreme sports is one of the most promising areas for future applied studies [45]. In extreme sports, CU may be used to assess the increase in lung fluid content [36]. PE can affect participants of particular extreme sports, such as the one that are exposed to hypo- and/or hyperbaric condition, such as climbers exposed to high altitude (above 4000 m), and divers exposed to a depth of −100 m under the sea level [47]. In these settings, PE may occur as a result of a combination of hemodynamic, mechanical, hypoxemic, and biochemical mechanisms [47]. However, also in the normal environment, ultraendurance extreme sports may cause lung tissue and microvascular endothelial damage, as a consequence of thermal, hormonal, and metabolic stress [36].

After Ironman race (which consists of swimming required for a distance equal to 3.86 km, riding a bike for a distance equal to 180 km, and running a marathon), CU detected a significant increase of B-lines. This was associated with a decrease in spirometric indices as well as in ventilatory performance [36]. In some cases, the transient subclinical increase in lung fluid content may represent a paraphysiological adaptation, but in others, it may represent an important marker of individual vulnerability to potentially life-threatening PE [47].

### 4.8. Devices and Future Advancements

Recently, the development of portable ultrasound for commercial use has delivered two main types of products [18]. The first one is represented by the portable devices, which also include power supply and case. These devices are easy to be transported and relatively small. They usually fit in a backpack such as the Vscan (GE, Chicago, IL), Lumify (Philips, Andover, MA), and iViz (SonoSite, Bothell, WA).

These systems are also able to transfer images wireless or via text messages or e-mail. Secondly, the other types of devices are the total wireless ultrasound technologies, which are receiving the images directly on the connected smartphone. Some examples are Clarius (Burnaby, Canada) and Signostics (Bothell, WA) [18].

Despite having new accurate devices able to be used in multiple ways, which are light and easy to be moved, yet there is no CU specifically designed for the extreme setting, currently commercially available. CU designed for the extreme setting should be waterproof, given the wide range of water sports. They should be resistant to cold conditions, in order to be used, for example, in sports performed on snow, as well as to strong vibrations and bright lights. Today, a totally waterproof ultrasound device is still lacking. Water can easily damage the electronic components of the device and the batteries [18]. In sea environments, the saltiness of the water can also be a crucial damaging component. Therefore, it would be worth to develop water-resistant ultrasounds for the future. The new extreme setting CU device should also be designed “bulletproof” for sands and dust, elements often present in extreme settings (e.g., deserts). At high altitude, with low atmospheric pressure and cold, the ultrasound devices can report issues at spinning hard drives. Consequently, it is best to use a system with a solid memory system and without moving components. It has been described before that the immersion of a transduction in warm water was a successful countermeasure for maintaining the functioning of the system [56]. In cold environments, the battery duration can be diminished; as a result, a new way to recharge the device should be developed. On the other hand, when the devices are exposed to heat, a special cooling system should be implemented.

As stated in previous studies, looking at a monitor full sole can be extremely difficult and disturbing for the clinicians [18]. This can be even worse when the screen is reflecting the sun or the snow in extreme settings. For this reason, it may be worth considering the screen-covering tools. The covering tools could range from a solid screen cover to small curtains to cover the operator and the screen. However, which material to use and how to build it, in order also not to overheat the physician and the devices, still remain to be explored. Watercrafts, helicopters, and other vehicles used to rescue the patients are often releasing high vibrations [18]. Reducing or diminishing the movements from the ultrasound scan is crucial to obtain good quality images. However, this can be very challenging in rescue vehicles normally used for remote areas. The development of specific vibration insulated litters could solve this problem. Ergonomics should also be considered.

### 4.9. Training/Guidelines and Future Perspectives

Aside from the technological advancements, it is essential that every team member, for example, of rescue teams adopting CU, is receiving the adequate CU trainings. The evidence-based application for using CU as a standard of care has greatly increased and with it now the efforts to integrate focused CU training into medical school curricula [57].

Multiple studies have shown that it is possible to learn the basic components of the CU with training programs lasting between two hours and four months to a number of supervised scans between 20 and 80 [58, 59]. For example, when considering medical students, a course, requiring generally 5 to 30 hours in total commitment over several weeks, was able to provide the key components of CU such as terminology, knobology, and image acquisition [57]. Given the technological advancement also in the trainings, CU education should become a routine for physicians of all kinds. Indeed, presently, CU can also be touched through the use of virtual reality. Heer [60] has previously demonstrated that virtual reality can easily facilitate students to learn how to perform CU and to read the images. For example, virtual reality programs are able to enhance the use of a simulator when teaching intraoperative transesophageal echocardiography (TEE). In such studies, it was indicated that the use of virtual reality and simulators led to a superior teaching method [61].

The time required to achieve basic competency in TEE was shortened than that with a curriculum that relies solely on experience with actual patients. Given that such technologies can easily be integrated during the courses, teaching ultrasound should no longer start from exclusive theoretical basis. In addition to this, the use of virtual reality could standardize the learning curriculum of CU, also across multiple countries [61, 62]. Virtual reality for teaching CU can enhance performances by providing repetitive training on selected cases of increasing difficulties, as well as including controlled exposure to rare cases as previously described [60–62]. Nevertheless, yet the integration of such courses is still in its primary phases. On the other hand, CU for extreme settings should also be touched to nonmedically trained team members. Specific studies about the exact time needed to train and different levels of training for the normal population are still missing. It is still unclear how much time is required to be able to perform the best possible imaging recording with basic medical knowledge. What is known is that CU teaching should incorporate different levels of training [29]. If these trainings were well developed and available to all, the usage of CU in extreme settings may increase.

Healthcare professionals could also follow up the patient with the use of telemedicine. A study presented by Strode [28] has demonstrated that, with telemedicine, it is possible to place critical patient information one step closer to a team of expert, from a remote setting, such as during combat triage or in extreme remote areas to a hospital. With these regards, the telemedicine examples provided by the past space missions [53] could represent the first step to create new telemedicine programs on Earth for CU in extreme settings.

Specific guidelines for CU should be implemented for extreme settings and with regard to specific conditions. The power of such a tool relies within the precision of the operators; as a result, the examinations must be performed accurately.

## 5. Conclusions

This review presented different usages of CU in multiple extreme environments and settings. Despite the usage of CU is well known for certain extreme environments such as for the military, it is still in a developmental phase for others such as in extreme sports. This review showed the versatility of CU and how it has been successfully implemented for treating multiple respiratory and chest conditions. Despite the technological improvements presented, it is clear that a specific CU device for the extreme setting is still missing. Suggestions on new potential aspects to consider in the design and development of specific CU for extreme conditions were also reported. Lastly, the usages of CU can only improve and increase with a parallel rise in its training. For this reason, an overview on trainings for clinicians and nonclinicians was also debated. With these examples, the usage of CU is encouraged for the near future for all extreme settings.

## Figures and Tables

**Table 1:** Applications of CU in extreme settings according to the literature.

Authors	Kind of study	Number of subjects	Setting	Condition assessed
Farrow [26]	*Pilot study*	19	UNMIL Hospital in Dili, East Timor, or with 3rd Health Support Battalion (3H SB)	Hemothorax and hemopericardium
Cremona et al. [27]	*Prospective study*	262	Monte Rosa mountain (4559 m)	PE
Strode et al. [28]	*Original research*	5	U.S. Army Combat Support Hospital, remotely placed in the field 20 miles from tertiary care	Pericardial effusion
Foale et al. [29]	*Original research*	1	International Space Station (ISS)	Normal anatomy
Fagenholz et al. [30]	*Original research*	11	Himalayan Rescue Association Clinic, Pheriche, Nepal (4240 m)	PE
Dean et al. [31]	*Original research*	3	Aftermath of Hurricane Stan (out of total 139 US examinations in 9 days)	Pleura and lung injuries
Frassi et al. [32]	*Prospective study*	31	International Apnea Diving Championship, Sharm El Sheikh, Egypt	PE
Otto et al. [33]	*Brief report*	2	Advanced Base Camp on Mount Everest (6400 m)	PE
Madill [34]	*Case report*	1	Forward operating base in combat scenario, Afghanistan	Pneumothorax
Pratali et al. [35]	*Observational study*	18	Trekking Gokyo and Khumbu Valley, Kathmandu (1350 m), Namche Bazaar (3440 m), Gokyo (4790 m), and Gorak Shep (5130 m), Nepal	PE
Pingitore et al. [36]	*Original research*	31	Ironman Triathlon (3.8 km swimming + 180 km cycling + 42 km running); Pisa, Italy (30 m above the sea level)	PE
Boussuges et al. [37]	*Original research*	30	Apnea diving competition in the Mediterrenean Sea (autumn and winter)	PE
Shorter and Macias [38]	*Retrospective observational*	6	7-Richter magnitude earthquake	Hemodynamic monitoring, PE, consolidation, atelectasis, and lung recruitment
Yin et al. [39]	*Retrospective analysis*	97	Lushan earthquake	Hemodynamic monitoring, PE, consolidation, atelectasis, and lung recruitment

PE = pulmonary edema.

**Table 2:** Conditions for which the use of CU has been proposed in the scientific literature.

Interesting contribution to diagnosis	Ultrasonographic method	Signs	Treatment
Pericardial effusion [28]	*Direct visualization*	Hypoanechoic layer posterior to the heart	Monitoring
Pericardial tamponade [17]	*Repeated assessment of pericardial effusion*	50% IVC change in diameter during inspiration (sensitive/nonspecific)/RA collapse that exceeds one-third of cardiac cycle is nearly 100% sensitive and specific/RV diastolic collapse (nonsensitive)/inspiratory septal shift is not specific	Ultrasound-guided long-needle periocardiocentesis
Intravascular volume depletion estimation [40]	*Measurement of the diameter of the IVC just below the diaphragm*	cIVC (IVCmax − IVCmin)/IVCmax >42%	Follow-up after fluid administration
PE [36, 41]	*Assessment of lung sonographic artifacts*	Presence of B-line artifacts: absence of A-line artifacts	Monitoring
Pneumothorax [34]	*Lung sliding detection*	Absence of lung sliding and lung points	To guide decompression of the correct pleural space
Pleural effusion [25]	*Direct visualization*	Hypoanechoic layer of polygonal area under the pleural line	Chest tube placement and verification
Pulmonary contusion [25]	*Direct visualization*	Lung hepatization: B-lines and shred sign	Monitoring
Soft tissue infection of the chest wall [18]	*Direct visualization*	Cellulitis has cobblestone appearance/abscesses are complex structures	Guide planning for the best location for incision and drainage
Bony thoracic cage fractures	*Direct visualization of the fracture and evaluation of complications (*e.g., *pneumothorax, pleural effusion, lung contusion, and hematoma of the chest wall)*	Chimney phenomenon, interruption of cortical bone, and soft tissue hematoma	

IVC: inferior vena cava; RV: right ventricle; RA: right atrium; cIVC: inferior vena cava collapsibility index; IVCmax: inferior vena cava maximum diameter; IVCmin: inferior vena cava minimum diameter; PE: pulmonary edema.

## References

[1] Littlejohn L. F. (2017). Treatment of thoracic trauma: lessons from the battlefield adapted to all austere environments. *Wilderness & Environmental Medicine*.

[2] Ma O. J., Norvell J. G., Subramanian S. (2007). Ultrasound applications in mass casualties and extreme environments. *Critical Care Medicine*.

[3] Sholl J. M., Curcio E. P. (2004). An introduction to wilderness medicine. *Emergency Medicine Clinics of North America*.

[4] Sward D. G., Bennett B. L. (2014). Wilderness medicine. *World Journal of Emergency Medicine*.

[5] Galvagno S. M., Mabry R. L., Maddry J. (2018). Measuring US Army medical evacuation: metrics for performance improvement. *Journal of Trauma and Acute Care Surgery*.

[6] Richards R., Awrey J. M., Medeiros S. E., McGahan J. P. (2017). Color and power Doppler sonography for pneumothorax detection. *Journal of Ultrasound in Medicine*.

[7] Hindle E. M., Henning J. D. (2014). Critical care at extremes of temperature: effects on patients, staff and equipment. *Journal of the Royal Army Medical Corps*.

[8] Feletti F., Aliverti A., Henjum M., Tarabini M., Brymer E. (2017). Incidents and injuries in foot-launched flying extreme sports. *Aerospace Medicine and Human Performance*.

[9] Feletti F. (2017). Preface. *Extreme Sports Medicine*.

[10] RedBull.com Team (2013). *Final findings of the Red Bull Stratos Scientific Summit*.

[11] Strat Ex (2015). *Alan Eustace and the Paragon StratEx Team Make Stratospheric Exploration History at over 135,000 Feet*.

[12] Rossati A. (2017). Global warming and its health impact. *International Journal of Occupational and Environmental Medicine*.

[13] Wongwaisayawan S., Suwannanon R., Sawatmongkorngul S., Kaewlai R. (2016). Emergency thoracic US: the essentials. *Radiographics*.

[14] Corcoran F., Bystrzycki A., Masud S., Mazur S. M., Wise D., Harris T. (2015). Ultrasound in pre-hospital trauma care. *Trauma*.

[15] Lichtenstein D. A. (2015). BLUE-protocol and FALLS-protocol: two applications of lung ultrasound in the critically ill. *Chest*.

[16] Melnick E. R. (2011). Ultrasound in emergency medicine portable ultrasound for remote environments, part I. *Feasibility of Field Deployment*.

[17] Russell C. T. C., Crawford P. F. (2013). Ultrasound in the austere environment: a review of the history, indications, and specifications. *Military Medicine*.

[18] Gharahbaghian L., Anderson K. L., Lobo V., Huang R. W., Poffenberger C. M., Nguyen P. D. (2017). Point-of-care ultrasound in austere environments: a complete review of its utilization, pitfalls, and technique for common applications in austere settings. *Emergency Medicine Clinics of North America*.

[19] Livingston D. H., Hauser C. J., Moore E. E., Feliciano D. V., Mattox K. I. (2003). Trauma to the chest wall and lung. *Trauma*.

[20] Blaivas M., Kuhn W., Reynolds B. (2005). Change in differential diagnosis and patient management with the use of portable ultrasound in a remote setting. *Wilderness & Environmental Medicine*.

[21] Gardelli G., Feletti F., Nanni A., Mughetti M., Piraccini A., Zompatori M. (2012). Chest ultrasonography in the ICU. *Respiratory Care*.

[22] Yousefifard M., Baikpour M., Ghelichkhani P. (2016). Screening performance characteristic of ultrasonography and radiography in detection of pleural effusion; a meta-analysis. *Emergency*.

[23] Kirkpatrick W., Sirois M., Lupland K. B. (2004). Hand-held thoracic sonography for detecting post-traumatic pneumothoraces: the Extended Focused Assessment with Sonography for Trauma (EFAST). *Journal of Trauma: Injury, Infection, and Critical Care*.

[24] Zanobetti M., Coppa A., Nazerian P. (2015). Chest abdominal-focused assessment sonography for trauma during the primary survey in the emergency department: the CA-FAST protocol. *European Journal of Trauma and Emergency Surgery*.

[25] Galdamez L. A., Clark J. B., Antonsen E. L. (2017). Point-of-care ultrasound utility and potential for high altitude crew recovery missions. *Aerospace Medicine and Human Performance*.

[26] Farrow G. B. (2001). Portable ultrasound on deployment: a pilot study. *ADF Health*.

[27] Cremona G., Asnaghi R., Baderna P. (2002). Pulmonary extravascular fluid accumulation in recreational climbers: a prospective study. *The Lancet*.

[28] Strode C. A., Rubal B. J., Gerhardt R. T. (2003). Satellite and mobile wireless transmission of focused assessment with sonography in trauma. *Academic Emergency Medicine*.

[29] Foale C. M., Kaleri A. Y., Sargsyan A. E. (2005). Diagnostic instrumentation aboard ISS: just-in-time training for non-physician crewmembers. *Aviation, Space, and Environmental Medicine*.

[30] Fagenholz P. J., Gutman J. A., Murray A. F., Harris N. S. (2007). Treatment of high altitude pulmonary edema at 4240 m in Nepal. *High Altitude Medicine & Biology*.

[31] Dean A. J., Ku B. S., Zeserson E. M. (2007). The utility of handheld ultrasound in an austere medical setting in Guatemala after a natural disaster. *American Journal of Disaster Medicine*.

[32] Frassi F., Pingitore A., Cialoni D., Picano E. (2008). Chest sonography detects lung water accumulation in healthy elite apnea divers. *Journal of the American Society of Echocardiography*.

[33] Otto C., Hamilton D. R., Levine B. D. (2009). Into thin air: extreme ultrasound on Mt Everest. *Wilderness & Environmental Medicine*.

[34] Madill J. J. (2010). In-flight thoracic ultrasound detection of pneumothorax in combat. *Journal of Emergency Medicine*.

[35] Pratali L., Cavana M., Sicari R., Picano E. (2010). Frequent subclinical high-altitude pulmonary edema detected by chest sonography as ultrasound lung comets in recreational climbers. *Critical Care Medicine*.

[36] Pingitore A., Garbella E., Piaggi P. (2011). Early subclinical increase in pulmonary water content in athletes performing sustained heavy exercise at sea level: ultrasound lung comet-tail evidence. *American Journal of Physiology-Heart and Circulatory Physiology*.

[37] Boussuges A., Coulange M., Bessereau J. (2011). Ultrasound lung comets induced by repeated breath-hold diving, a study in underwater fishermen. *Scandinavian Journal of Medicine & Science in Sports*.

[38] Shorter M., Macias D. J. (2012). Portable handheld ultrasound in austere environments: use in the Haiti disaster. *Prehospital and Disaster Medicine*.

[39] Yin W., Zhou R., Wu H. (2014). Value of focused critical ultrasound in the treatment of critical patients in Lushan earthquake. *Zhonghua Yi Xue Za Zhi*.

[40] Airapetian N., Maizel J., Alyamani O. (2015). Does inferior vena cava respiratory variability predict fluid responsiveness in spontaneously breathing patients?. *Critical Care*.

[41] Wimalasena Y., Windsor J., Edsell M. (2013). Using ultrasound lung comets in the diagnosis of high altitude pulmonary edema: fact or fiction?. *Wilderness & Environmental Medicine*.

[42] Yamaji F., Okada H., Nakajima Y. (2017). Blunt cardiac injury due to trauma associated with snowboarding: a case report. *Journal of Medical Case Reports*.

[43] Lee R. K., Griffith J. F., Ng A. W., Sitt J. C. (2015). Sonography of the chest wall: a pictorial essay. *Journal of Clinical Ultrasound*.

[44] Ferre R. M., Johnson J., Hall B. (2017). *Military and Tactical Ultrasound*.

[45] Evans C. S., Harris N. S. (2012). Ultrasound and ski resort clinics: mapping out the potential benefits. *Wilderness & Environmental Medicine*.

[46] Ellington L. E., Gilman R. H., Chavez M. A. (2017). Lung ultrasound as a diagnostic tool for radiographically-confirmed pneumonia in low resource settings. *Respiratory Medicine*.

[47] Garbella E., Catapano G., Pratali L., Pingitore A. (2011). Pulmonary edema in healthy subjects in extreme conditions. *Pulmonary Medicine*.

[48] Chun R., Kirkpatrick A. W., Sirois M. (2004). Where’s the tube? Evaluation of hand-held ultrasound in confirming endotracheal tube placement. *Prehospital and Disaster Medicine*.

[49] Richards J. R., McGahan J. P. (2017). Focused Assessment with Sonography in Trauma (FAST) in 2017: what radiologists can learn. *Radiology*.

[50] Nations J. A., Browning R. F. (2011). Battlefield applications for handheld ultrasound. *Ultrasound Quarterly*.

[51] Stawicki S. P., Howard J. M., Pryor J. P., Bahner D. P., Whitmill M. L., Dean A. J. (2010). Portable ultrasonography in mass casualty incidents: the CAVEAT examination. *World Journal of Orthopedics*.

[52] Huber D. G., Gulledge J. (2011). *Extreme Weather and Climate Change: Understanding the Link and Managing the Risk*.

[53] Law J., Macbeth P. B. (2011). Ultrasound: from earth to space. *McGill Journal of Medicine*.

[54] Hamilton D., Smart K., Melton S., Polk J. D., Johnson-Throop K. (2008). Autonomous medical care for exploration class space. *Journal of Trauma: Injury, Infection, and Critical Care*.

[55] Immonen T., Brymer E., Orth D. (2017). Understanding action and adventure sports participation-an ecological dynamics perspective. *Sports Medicine-Open*.

[56] Fagenholz P. J., Murray A. F., Noble V. E. (2012). Ultrasound for high altitude research. *Ultrasound in Medicine & Biology*.

[57] Bahner D. P., Royall N. A. (2013). Advanced ultrasound training for fourth-year medical students: a novel training program at The Ohio State University College of Medicine. *Academic Medicine*.

[58] Bedetti G., Gargani L., Corbisiero A. (2006). Evaluation of ultrasound lung comets by hand-held echocardiography. *Cardiovascular Ultrasound*.

[59] Flato U. A. P., Guimaraes H. P., Petisco G. (2015). Use of lung ultrasonography in the detection of pneumothorax among medical students and emergency physicians. *Critical Care*.

[60] Heer I. M., Middendorf K., Müller-Egloff S., Dugas M., Strauss A. (2004). Ultrasound training: the virtual patient. *Ultrasound in Obstetrics and Gynecology*.

[61] Jelacic A., Bowdle K., Togashi K., Vonhomeyer P. (2013). The use of TEE simulation in teaching basic echocardiography skills to senior anesthesiology residents. *Journal of Cardiothoracic and Vascular Anesthesia*.

[62] Konge L., Albrecht-Beste E., Nielsen M. B. (2014). Virtual-reality simulation-based training in ultrasound. *Ultraschall in der Medizin-European Journal of Ultrasound*.

